# Cerebellar anaplastic ganglioglioma in a septuagenarian

**DOI:** 10.1016/j.radcr.2024.01.007

**Published:** 2024-01-20

**Authors:** Andrew Waack, Alexander Luna, Jordan Norris, Noah King, Aastha Bhavsar, Jason Schroeder, Alastair Hoyt

**Affiliations:** Department of Surgery, Division of Neurosurgery, University of Toledo Medical Center, 3000 Arlington Ave Toledo, OH 43614, USA

**Keywords:** Cerebellum, Ganglioglioma, Rare, Advanced age, Posterior fossa

## Abstract

Ganglioglioma is a rare neoplasm most common in children and adolescents. It is typically located in the supratentorial compartment, with the temporal lobe being the most common tumor location. Anaplastic ganglioglioma is a WHO grade III ganglioglioma, a rare subtype accounting for a small minority of ganglioglioma cases. Posterior fossa anaplastic ganglioglioma in an adult is incredibly rare; only 3 prior cases have been reported. Only 1 adult anaplastic ganglioglioma in the cerebellum has been reported. We present the second reported adult cerebellar anaplastic ganglioglioma.

## Introduction

Ganglioglioma (GG) is a rare intracranial neoplasm composed of ganglion and glial cell types. GGs comprise 0.4%-1.3% of intracranial neoplasms and predominately affect young people, with most GGs occurring in the second decade of life [1]. Anaplastic ganglioglioma (AGG) is a grade III ganglioglioma. It is a rare subtype, representing approximately 1%-10% of GGs [2,3]. AGG can arise de novo or secondary to malignant transformation of low grade GG [3,4]. While GG typically carries a good prognosis with a two-year survival rate of 90%, AGG has a poorer prognosis with a 2-year survival rate of approximately 40% [4]. Both GG and AGG most often occur in the temporal lobe [5]. Reports of anaplastic posterior fossa GG are exceptionally rare, especially in advanced age [6,7].

We present a case of cerebellar AGG in a 75-year-old female. To our knowledge, this is the first report of a cerebellar AGG patient aged more than 40 years [6].

## Case description

**Fig. 1:**
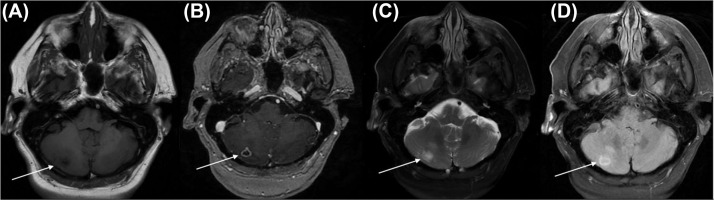
Imaging on initial presentation (A) T1 weighted MR image (B) T1 weighted MR image with contrast (C) T2 weighted MR image with fat suppression (D) FLAIR MR image. White arrows denote the right cerebellar mass.

A 75-year-old female presented to the emergency room with acute left-sided weakness and was subsequently hospitalized. A brain MRI demonstrated a small right lacunar stroke. Other findings included a right parafalcine meningioma which had been present and stable for many years on imaging, as well as a new ring-enhancing mass of the right cerebellum (Fig. 1). The mass was fundamentally different in appearance from the meningioma, raising suspicion for underlying unknown malignancy. Computed tomography of the chest, abdomen, and pelvis showed no evidence of malignancy. It was elected to observe the incidentally discovered lesion.

**Fig. 2:**
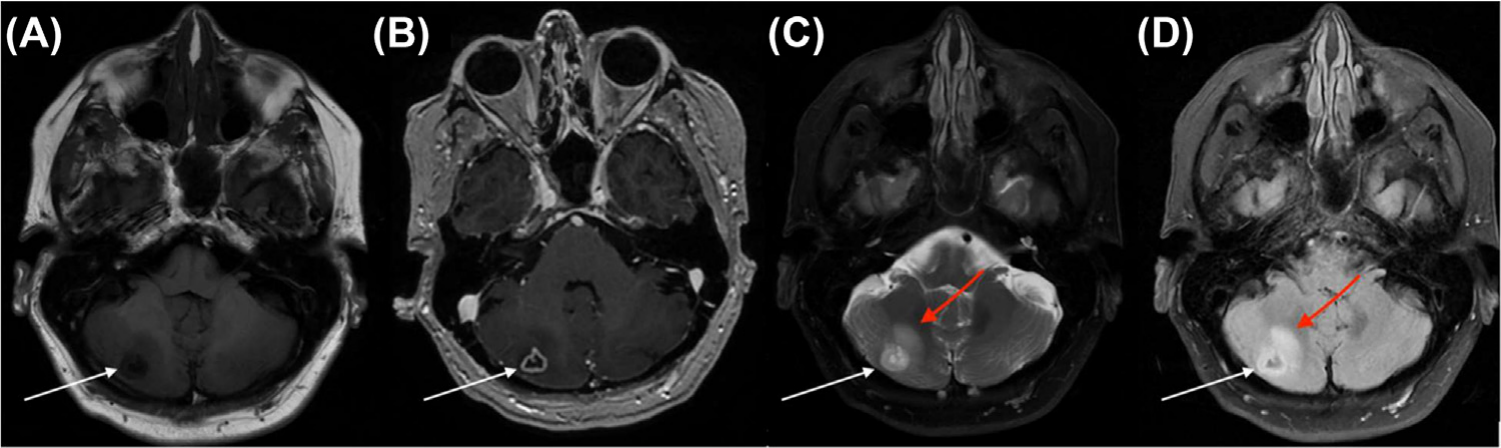
Follow up imaging 6 weeks after initial presentation (A) T1 weighted MR image (B) T1 weighted MR image with contrast (C) T2 weighted MR image with fat suppression, red arrow demonstrates interval increased signal adjacent to the lesion and (D) FLAIR MR image, red arrow demonstrates interval increased signal adjacent to the lesion. White arrows again denote the right cerebellar mass, with stable lesion morphology from imaging 6 weeks prior.

At an outpatient clinic visit 6 weeks later, the patient reported occasional posterior headaches and decreasing weakness. She denied nausea, vomiting, blurred vision, and diplopia. She was ambulatory with a rolling walker and had been undergoing physical and occupational therapy. Physical examination showed was grossly stable, with minimal residual left-sided weakness and no abnormalities on cerebellar testing. Repeat MR imaging revealed stable lesion morphology but increased signal was seen adjacent to the lesion on T2 and FLAIR sequences. There was no evidence of central restricted diffusion, and no additional lesions were identified (Fig. 2).

**Fig. 3:**
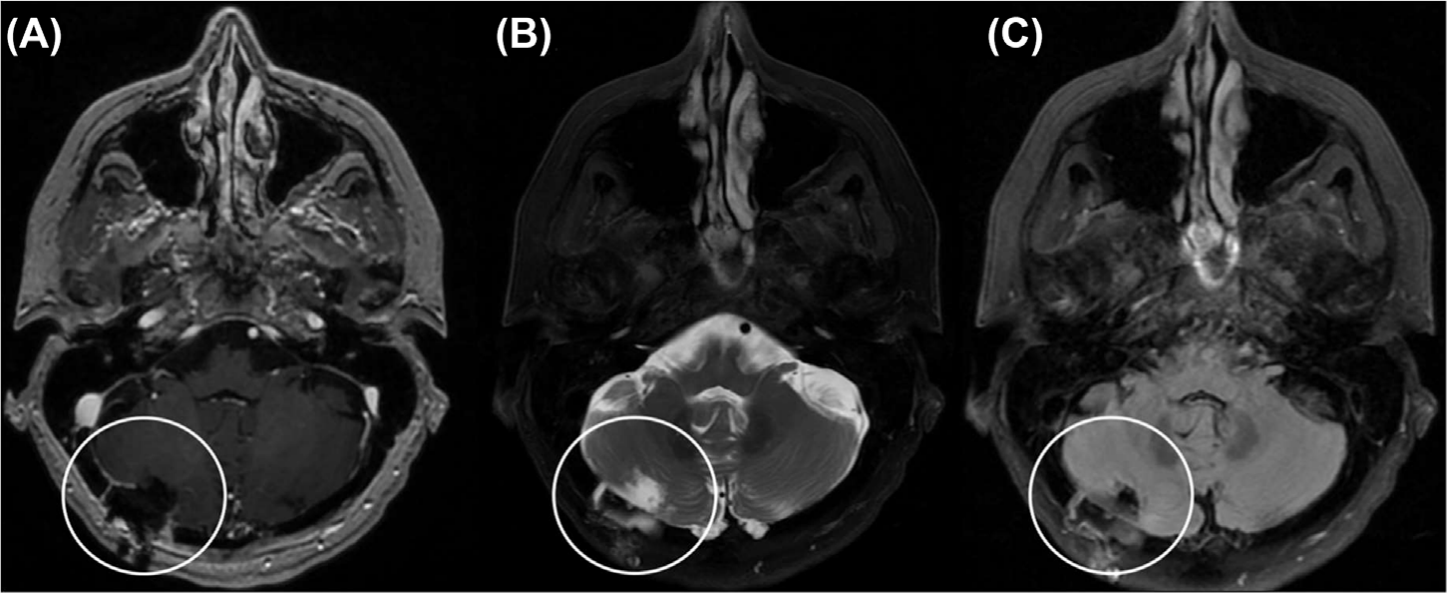
Postoperative imaging demonstrating gross total resection and post-craniotomy changes (encircled in white). (A) T1 weighted MR image with contrast (B)T2 weighted image with fat suppression and (C) FLAIR MR image.

Given progression of T2 signal surrounding the lesion, surgery for excisional biopsy was recommended to the patient, who elected to proceed.  An uneventful right posterior fossa craniotomy for excisional biopsy of the lesion was performed, which the patient tolerated well. Postoperative MRI demonstrated a gross total resection, and the postoperative course was uneventful (Fig. 3). Frozen pathology confirmed abnormal tissue but could not establish a diagnosis. The patient was discharged home on the first postoperative day without new deficits.

Expert neuropathology consultation revealed a ganglioglioma with anaplastic features. The neoplastic cells included some cells with prominent nucleoli as well as binucleated cells. Other neoplastic cells were characterized by elongated nuclei with glial cytology. Immunohistochemistry was positive for CD34, weakly positive for NeuN, and some cells were immunoreactive for synaptophysin. Ki67 labeled many cells constituting the microvascular hyperplasia, with the parenchymal tumor cells showing a labelling index of about 5%. P53 showed weak to moderate intensity staining about 50% of the tumor cells. Oligodendrocyte transcription factor 2 and glial fibrillary acidic protein highlighted the glial component of the tumor. Alpha thalassemia/mental retardation syndrome X-linked (ATRX) gene was positive and IDH1R132H was negative. IDH1/2 sequencing was negative for a mutation and BRAFV600E mutation analysis was also negative. EGFR was not amplified by FISH, and MGMT was not hypermethylated.

Postoperatively, the patient underwent intensity-modulated radiation therapy of 40 gray in 15 fractions. She was offered chemotherapy with temozolomide but declined treatment. MRI imaging slightly less than 6 months postoperatively revealed no evidence of recurrent diseases. Continued observation is planned.

## Discussion

Gangliogliomas (GGs) are neuronal-glial admixtures. They are most frequently seen in the temporal lobes or frontal lobes during childhood or adolescence [1,5,8]. In the temporal lobes, GGs are highly epileptogenic, with seizures occurring in up to 42% of patients [9]. Additional symptoms of temporal GG include nausea, headache, papillary edema, diplopia, and hemiplegia [8,9]. Although uncommon, GGs can occur in other locations throughout the neuraxis, including the posterior fossa [8]. Compared to supratentorial GGs, posterior fossa GGs typically demonstrate larger dimensions, fewer cystic changes, and cerebellar atrophy [1]. Additionally, posterior fossa GGs may cause obstructive hydrocephalus and cranial nerve palsies [1,6]. Like low grade GG, AGG is typically located in the supratentorial compartment, and the posterior fossa is a rare location for AGG [1,10].

Most GGs are diagnosed in the second decade of life and are uncommon in adults over the age of 25 [1,11]. Additionally, GGs in older patients are commonly higher grade than those occurring in younger patients [11]. GGs account for approximately 1% of CNS neoplasms, and AGG comprises up to 10% of GGs; the annual incidence of AGG is 0.02 per million [2,3].

The infratentorial compartment is a rare location for AGG, and advanced age is an extremely rarely affected demographic. To date, only 3 cases of AGG have been reported in the posterior fossa of an adult: 1 in the cerebellum [6] and 2 in the brainstem [7,8]. Toledo-Gonzalez et al. reported a case of AGG in the brainstem of a 33 year old male, while Han et al. reported a case of AGG in the brainstem of a 45 year old male, and Bouli et al. reported a case of AGG in the cerebellum of a 40-year-old male [6], [7], [8]. Bouli et al.’s [6] case represents the only reported adult, cerebellar AGG. The patient presented in this report is the oldest known individual to harbor a posterior fossa AGG, and is only the second reported with adult cerebellar AGG [6], [7], [8].

GG is diagnosed through radiological imaging, histological analysis, and molecular techniques. Various imaging modalities have been used to describe AGG, which often present on imaging as cystic, heterogenous lesions [2,4]. AGG typically presents as hypointense to isointense on T1- weighted magnetic resonance imaging (MRI) [2,7,10]. The lesions may display a patchy enhancement pattern with gadolinium contrast and are hyperintense on T2-weighted and fluid attenuated inversion recovery MR imaging [2,7,10]. The high cellularity characteristic of AGG reduces the apparent diffusion coefficient in diffusion weighted imaging compared to low grade GG [7]. On magnetic resonance spectroscopy, AGG demonstrates elevated choline, glutamate, and myoinositol [7,10]. T2-weighted magnetic resonance spectroscopy, apparent diffusion coefficient, and MRS imaging modalities can help differentiate low grade GG from AGG [7]. However, radiological imaging alone is insufficient for discriminating GGs from AGG or other brain tumor types [12]; histological workup must be performed along with radiological imaging to diagnose AGG.

Histologically, GGs present as heterogenous admixtures of dysplastic glial and ganglion cells. There is great variability in histological appearance [13,14]. Immunohistochemistry and molecular techniques may assist in accurately identifying GG [2]. GGs demonstrate glial fibrillary acidic protein, S-100, and vimentin positivity in the glial component and synaptophysin, MAP 2, NeuN and neurofilament positivity in the neural components [14]. AGG can be histologically differentiated from GG by microvascular proliferation, glial and neuronal mitoses, necrotic foci, hypercellularity, and cellular pleomorphism [2,4,13]. Although absent in the reported case, a high percentage of AGGs display BRAF V600E mutations in both the glial and neuronal cell populations, suggesting these cells arise from a common precursor and providing a possible therapeutic target [2,3].

The diagnosis of AGG is currently controversial in neuropathology. As pointed out in the WHO 2021 Diagnostic Guidelines for CNS tumors, much of the published literature regarding the histological diagnosis of AGG has been published prior to the widespread use of deep sequencing methods. This may have failed to adequately exclude high grade gliomas as possible diagnoses; if true, this would further decrease the incidence of the already extremely rare AGG, and it may also improve the reported unfavorable prognosis.

Due to the low frequency of reported AGG cases, no treatment protocol has been established [6]. Surgical resection is the preferred treatment for AGG, with extent of resection being the most important prognostic factor [6,10]. While low grade GG can be cured with total surgical resection, the outcomes remain poor when treating AGG with surgery alone [9,15]. The use of adjuvant radiation and chemotherapy has been reported for AGG, although these modalities do not improve survival [6,10]. However, radiation therapy does appear to improve local control rates [6,10]. Interestingly, vemurafenib, a selective BRAF inhibitor, has been proposed for AGGs with BRAF V600E mutations following positive outcomes in the use of melanoma brain metastases [6,9]. Even following surgical resection, often supplemented with adjuvant radiation and chemotherapy, the prognosis of AGG is poor, with a median survival of 27 months [6].

## Conclusion

We present the case of AGG located in the cerebellum of a septuagenarian. This is the second reported case of an AGG in the cerebellum of an adult and the only report of an infratentorial AGG in a person older than 40 [6].

## Patient consent

Patient consent was obtained.
